# Systematic Study on a Quantitative Analysis of Multicomponents by Single Marker (QAMS) Method for Simultaneous Determination of Eight Constituents in Pneumonia Mixture by UPLC-MS/MS

**DOI:** 10.1155/2021/8311588

**Published:** 2021-11-03

**Authors:** Haibo Zhang, Weina Xie, Jiangyun Liu, Xiaoqiang Xiang, Shilei Zhang, Junping Hu, Jianhua Yang

**Affiliations:** ^1^College of Pharmacy, Xinjiang Medical University, Urumqi 830011, China; ^2^Department of Pharmacy, The First Affiliated Hospital of Xinjiang Medical University, Urumqi 830011, China; ^3^College of Pharmaceutical Sciences, Soochow University, Suzhou 215123, China; ^4^Department of Clinical Pharmacy, School of Pharmacy, Fudan University, Shanghai 201203, China

## Abstract

Pneumonia mixture was formulated and is available to treat children acute pneumonia and acute bronchitis in our hospital for nearly forty years, but there are few studies of its quality evaluation or control. In this paper, a new strategy for quality evaluation of pneumonia mixture was explored and verified through qualitative and quantitative analyses of multicomponents by single marker (QAMS) by UPLC-MS/MS. Baicalein was selected as an internal reference, and the relative correction factors (RCFs) and the relative retention time (RRT) of (R, S)-goitrin, amygdalin, chlorogenic acid, pseudoephedrine hydrochloride, ephedrine hydrochloride, ammonium glycyrrhizinate, and baicalin were established. The robustness and durability of the QAMS method were investigated. RCF values calculated by the average (AVG) method and linear regression (LRG) method had good repeatability and were acceptable for quantitative analysis, and the RTT combined with the exact masses of precursor and fragment ions and their abundance could be adopted for accurately positioning the chromatographic peak of the eight constituents. The consistency and feasibility of the QAMS method were verified by comparing the contents of the seven components calculated by a classic and validated external standard method (ESM) with those of the QAMS method, which reduces analytical cost and time of detection and avoids the problem of the diversity and large quantity of reference standards. The results demonstrated that the QAMS method developed in this paper could provide a new, alternative, and promising method to comprehensively and effectively determine multicomponents and control the quality of pneumonia mixture or even a group of similar medicines.

## 1. Introduction

Pneumonia, usually caused by virus, bacteria, mycoplasma, chlamydia, or multiple pathogens, is a menace to the healthy growth of children around the world. The major pathogens are bacteria, including *Haemophilus influenzae*, *Staphylococcus aureus*, *Streptococcus pneumoniae*, *Moraxella catarrhalis*, *Escherichia coli*, and *Klebsiella pneumoniae*. The main clinical symptoms of the disease are cough, expectoration, asthma, and fever [1–3]. Pneumonia is a common pulmonary infectious disease with the highest incidence and mortality in Chinese children, causing serious consequences including oxygenation index descending, rapid breathing, or even death.

Western medicine in the treatment of bacterial pneumonia mainly uses antibiotics to remove the bacteria, reduce symptoms, and ameliorate the pathologic changes in the lung [4]. In recent years, because of pathogen changes and the increasing drug resistance rate, the rates of hospitalization and deaths of bacterial pneumonia in children remain stubbornly high [5–7]. A large body of evidence of evidence-based medicine shows that integrated traditional Chinese and western medicine is more effective in treating bacterial pneumonia [8–10].

Pneumonia mixture is a hospital preparation of The First Affiliated Hospital of Xinjiang Medical University, which was developed by clinicians in our hospital according to their experience of treatment of children's pneumonia, and Maxing Shigan decoction in Shanghan Lun. Pneumonia mixture consists of *Scutellaria baicalensis* Georgi., *Glycyrrhiza uralensis* Fisch., *Ephedra sinica* Stapf, *Isatis indigotica* Fort., Gypsum fibrosum, *Prunus armeniaca* L., *Phragmites communis* Trin., and *Lonicera japonica* Thunb. Pneumonia mixture achieves good effect in clearing lung, diminishing inflammation, and relieving cough and asthma with small side and remarkable effects, which win high public praise in patients. Pneumonia mixture has been used to treat children's acute pneumonia and acute bronchitis in our hospital for nearly forty years, which produces both social and economic benefit [11–13].

Modern pharmacological and clinical studies showed that each medicinal material in the prescription possessed a variety of biological activities, such as diminishing inflammation, antipyretic effect, expectorant effect, antimicrobial activity, and relieving cough and asthma [14–21]. It seems like the choice of those multiple compounds to be controlled in each drug is highly dependent on the drug composition, desired pharmacological effect, and the availability of the standards. In Chinese Pharmacopoeia [22], the index components of each medicinal material in the prescription for determination are pseudoephedrine hydrochloride, ephedrine hydrochloride, amygdalin, baicalein, baicalin, (R, S)-goitrin, chlorogenic acid, and ammonium glycyrrhizinate, which can be used as the quality control standards of pneumonia mixture.

There have been some reports on the quantification of pneumonia mixture using UV and HPLC [23–25]. However, the quality control of pneumonia mixture was evaluated only using a single index and ignored the other active ingredients, which was insufficient to provide the chemical information for quality assessment. With the development of modernization process of traditional Chinese medicines (TCMs), simultaneous determination of multicomponents for quality control of TCMs becomes more and more popular and acknowledged [26]. Since the effect of pneumonia mixture might result from the synergy of multiple components and their multitarget effects, a reliable, sensitive, and uncomplicated quantitative method based on the diverse constituents is needed to be developed to control the preparation quality better.

Multiple reference standards were used for the analysis of multiple components, as the normal external standard method (ESM). However, the application of external standard method was limited in some cases due to the diversity and large quantity of reference standards, some of which are expensive, unstable, or lacking. To some extent, ESM may have relatively low efficiency [27]. In contrast, a quantitative analysis of multicomponents via a single marker (QAMS) method could solve this problem. QAMS method utilizes one inexpensive, easily available, and common active component as internal reference standard (IRS) to calculate the relative correction factors (RCFs) among IRS and other components with response and concentration, and then to calculate the content of other components. For the difficulty to isolate chemical reference substance from TCM and the instabilities of it, QAMS method can reduce analytical cost and time of detection and avoid the problem of the diversity and large quantity of reference standards [28]. It has been reported that QAMS has been applied in the determination of Sanhuang Gypsum Soup, *Pyrrosia* species, notoginseng, Gastrodia Elata Tubers, and other botanical ingredients [29–32]. QAMS method has been also acknowledged by both United States Pharmacopeia (USP) and Chinese Pharmacopoeia (ChP) and successfully applied to quality control of herb extracts and botanical ingredients in both USP 30-NF25 and ChP 2015 edition (volume I) [33, 34]. The most commonly used analytical method combined with QAMS was developed using high performance liquid chromatography coupled with ultraviolet detection (HPLC-UV), and the different analytes usually have similar UV absorption features. The weak UV absorption in detection wavelength with different chemical structures, longer detection time, identification of target analytes only by the relative retention time, and complex background interference restricted the application of HPLC-UV in QAMS. Liquid chromatography coupled with mass spectrometry (LC-MS) has attracted increasing attention due to its enhanced accuracy, reliability, sensitivity, rapidity, specificity, and reduced cost of analysis without consideration of the UV absorption features of different analytes. In addition, the position of the analytes can be identified and located by the relative retention time combined with the exact masses of precursor and fragment ions and their abundance without reference standards in this method [35].

In this study, eight active components, pseudoephedrine hydrochloride, ephedrine hydrochloride, amygdalin, baicalein, baicalin, (R, S)-goitrin, chlorogenic acid, and ammonium glycyrrhizinate, were simultaneously assayed by developed and validated ESM and QAMS method by using ultraperformance liquid chromatography-mass spectrometry (UPLC-MS/MS). In QAMS method, baicalein was used as the IRS, because it is easily available. Furthermore, the results of the QAMS method were compared with those of the ESM to verify the accuracy of QAMS method. QAMS method could potentially be applied for the quantitative quality of pneumonia mixture. Our findings offer a suitable and efficient approach for assessing the quality of pneumonia mixture or even a group of similar medicines.

## 2. Materials and Methods

### 2.1. Chemicals and Reagents

**Figure 1:**
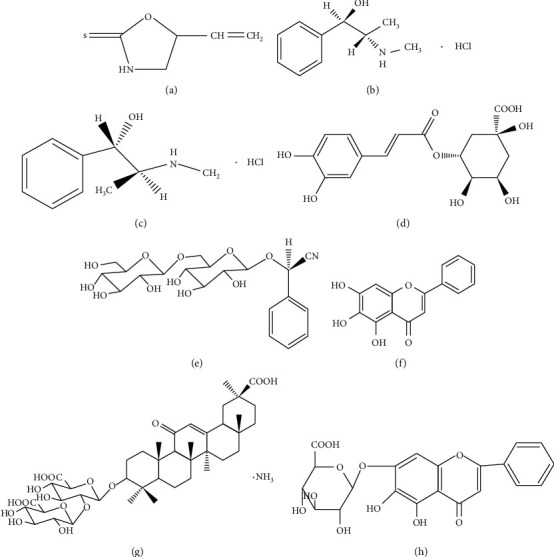
The chemical structures of the investigated compounds: (a) (R, S)-goitrin, (b) ephedrine hydrochloride, (c) pseudoephedrine hydrochloride, (d) chlorogenic acid, (e) amygdalin, (f) baicalein, (g) ammonium glycyrrhizinate, and (h) baicalin.

Pneumonia mixture was provided by the Department of Pharmacy, the First Affiliated Hospital of Xinjiang Medical University, Urumqi, China. Ephedrine hydrochloride (purity = 100%) and pseudoephedrine hydrochloride (purity = 99.8%) were obtained from National Institutes for Food and Drug Control, Beijing, China. Amygdalin (purity ≥ 98%), baicalein (purity ≥ 98%), baicalin (purity ≥ 98%), (R, S)-goitrin (purity ≥ 98%), chlorogenic acid (purity ≥ 98%), and ammonium glycyrrhizinate (purity ≥ 98%) were purchased from Shanghai Yuanye Bio-Technology Co., Ltd., Shanghai, China. The structures of those eight active components are shown in Figure 1 [36]. HPLC-grade methanol (MeOH) was procured from Fisher Scientific, Fair Lawn, NJ, USA. HPLC-grade formic acid was obtained from Shanghai Macklin Biochemical Co., Ltd., Shanghai, China. Ultrapure water used throughout the experiments was prepared by a PURELAB Chorus Ultrapure water purification system (ELGA, High Wycombe, UK).

### 2.2. Preparation of Standard Solutions

The stock solutions of pseudoephedrine hydrochloride, ephedrine hydrochloride, amygdalin, baicalein, baicalin, (R, S)-goitrin, chlorogenic acid, and ammonium glycyrrhizinate were prepared in methanol. Working standard solutions were prepared from stock solutions by diluting with 0.1% formic acid/methanol (95 : 5, v/v). All the stock solutions and the standard working solutions were stored at 4 °C. The calibration curve was plotted in the range of 0.098–100 ng/mL for (R, S)-goitrin, 1.56–1600 ng/mL for amygdalin, 0.20–840 ng/mL for chlorogenic acid, 0.056–460 ng/mL for pseudoephedrine hydrochloride, 0.032–260 ng/mL for ephedrine hydrochloride, 7.5–480 ng/mL for ammonium glycyrrhizinate, 1.82–465 ng/mL for baicalein, and 0.29–2400 ng/mL for baicalin.

### 2.3. Preparation of the Sample Solution

Pneumonia mixture was diluted 5000 times by 0.1% formic acid/methanol (95 : 5, v/v) and centrifuged at 12,000 rpm for 20 min at 4°C. A 5 *μ*l aliquot of the supernatant was injected into the UPLC-MS/MS system for analysis.

### 2.4. Liquid Chromatography and Mass Spectrometric Conditions

The chromatographic separations were carried out using a Waters Acquity UPLC I-Class system (Waters, Milford, USA). The separation was performed on an ACQUITY UPLC® BEH C18 column (2.1 × 50 mm, 1.7 *μ*m) with a linear gradient mobile phase consisting of methanol (phase A) and water containing 0.1% formic acid (phase B) at 0.4 mL/min. The gradient elution program was set as follows: 0–2.5 min (5% A), 2.5–7.0 min (5–85% A), 7.0–8.0 min (85–95% A), 8.0–9.0 min (95–5% A), and 9.0–10.0 min (5% A). The column temperature was maintained at 30°C and the autosampler was conditioned at 10°C.

Mass spectrometry analysis was performed on a Xevo TQ-S micro (Waters, Milford, USA) mass spectrometer. All the stock solutions were diluted to appropriate concentration with 0.1% formic acid/methanol (95 : 5, v/v) and infused into the mass spectrometer by using interactive fluidics system to optimize the (MRM) parameters, individually. The influences of positive and negative ion electrospray ionization modes on the intensity of the eight analytes were investigated. The parent ions were determined by MS scan, and daughter ions were selected by daughter scan for the subsequent transition. The data showed that positive ion electrospray ionization (ESI+) mode was better with higher sensitivity and efficiency. The optimized multiple reaction monitoring (MRM) parameters for the analytes, including transitions, cone, and collision voltage, are shown in Table S1. The other main working parameters for mass spectrometry were set as follows: the capillary voltage, 0.5 kV; desolvation temperature, 350 °C; source temperature, 150 °C; desolvation gas flow, 650 L/h. Argon was used as the collision gas. Dwell time was automatically set by MassLynx (Waters Corp., Milford, MA, USA). The MS/MS scan spectra of the eight analytes are shown in Figure S1. The acquisition and processing of data were performed using MassLynx (V4.1) software.

### 2.5. Method Validation

The UPLC-MS/MS method was validated in accordance with the USP32–NF27 < 1225 Validation of Compendial Procedures> and other related guidelines of CFDA [37, 38].

#### 2.5.1. Specificity

The method specificity was assessed by comparing the mixed standard and sample solutions, which were analyzed according to the method described in Section 2.4. There should be no interfering endogenous compounds observed at the retention times of the eight analytes.

#### 2.5.2. Linearity

Linear calibration curves were determined by a series of mixed standard solution diluents and established by plotting the peak area of the analytes (*y*-axis) versus the nominal concentrations of the analytes (*x*-axis) using a weighted (1/*x*) least-squares linear regression method.

#### 2.5.3. Precision

The precision of the established method was determined by measuring intra- and interday precision from analysis of the same mixed standard solution. Intraday precision for each compound was assessed by analyzing a mixed standard solution for six times in a single day and interday precision was performed by testing the same mixed standard solution on three consecutive days. The precision was assessed by relative standard deviation (RSD) of the peak area.

#### 2.5.4. Stability

To evaluate the stability of the sample, the sample solution was stored in an autosampler at 15 °C and then analyzed at 0, 2, 4, 6, 8, 10, and 12 h. The stability was evaluated by relative standard deviation (RSD) of the peak areas of each component at various time.

#### 2.5.5. Repeatability

To confirm the repeatability, six independent sample solutions were prepared and analyzed according to the method described in Section 2.4. Relative standard deviations (RSDs) of the peak areas of each component were calculated and used as parameters determining the repeatability of the method.

#### 2.5.6. Accuracy

The recovery was used to evaluate the accuracy of the method by standard addition method. The mixed standards with different concentration levels (high, medium, and low) were added into the certain amount of pneumonia mixture. The spiked samples were then extracted and analyzed by UPLC-MS/MS, and five replicate experiments were performed at each level. The recovery (%) was calculated using the following equation:

(1) $$\begin{array}{c} \text{recovery }\left(\text{\%}\right)=\left[\frac{\left(\text{amount found}-\text{original amount}\right)}{\text{amount spiked}}\right]\times 100\text{\%.} \end{array}$$

### 2.6. Theory of the QAMS Method

#### 2.6.1. Calculation of Relative Conversion Factors (RCFs)

*Average (AVG) Method*. [27, 28, 31, 39]. AVG method has been applied in most of the QAMS method-related studies, according to the principle that, within a concentration range, the response peak area of an analyte is linearly proportional to its concentration and their relations could be described with the following equation:

(2) $$\begin{array}{c} A=f•C, \end{array}$$

where *C* is the concentration of the analyte, *A* is the response peak area of the analyte, and *f* is the correction factor.

The value of *f* is a constant related to the detected substance and the sensitivity of the detector and could be described in the following equation:

(3) $$\begin{array}{c} f_{k}=\frac{A_{k}}{C_{k}}, \end{array}$$

where *A*_*k*_ is the peak area of the analyte and *C*_*k*_ is the concentration of the analyte.

One of those investigated components can be taken as internal reference standard (IRS, components “*s*“), and then the RCFs are calculated among IRS and other components via concentration and peak area of a series of mixed standard solution diluents in (4) in Section 2.5.2. The selected IRS has the following properties: low cost, high stability, high content, being easy to obtain, and significant pharmacological activities. Using the RCF, the concentration of multicomponents “*k*” in the samples can be calculated without reference substance according to the following equation:

(4) $$\begin{array}{c} f_{ks}=\frac{f_{k}}{f_{s}}=\frac{A_{k}/C_{k}}{A_{s}/C_{s}}, \end{array}$$

(5) $$\begin{array}{c} C_{K}=n\times \frac{1}{f_{ks}}\times C_{s}\times \frac{A_{K}}{A_{s}}, \end{array}$$

where *A*_*k*_ and *A*_*s*_ are the peak area of the analyte and the internal reference standard (IRS) in the standard solution, respectively. *C*_*k*_ and *C*_*s*_ are the concentration of the analyte and the IRS in the standard solution, respectively. *f*_*ks*_ is the average relative conversion factor of each analyte to IRS. *A*_*K*_ is the peak area of the analyte in the pneumonia mixture. *C*_*K*_ is the mass concentration of the analyte in the pneumonia mixture (mg/mL). *n* is the dilution multiple of pneumonia mixture in preparation of the sample solution.

*Linear Regression (LRG) Method* [29, 30, 35]. The selected internal reference standard in this method was the same as that in the AVG method. The RCFs were obtained for other components by calculating the ratios of the slopes of their calibration equations to that of IRS equation:

(6) $$\begin{array}{c} f_{ks}=\frac{a_{k}}{a_{s}}. \end{array}$$

The content of the measured component was calculated as follows:

(7) $$\begin{array}{c} C_{K}=n\times \frac{1}{f_{ks}}\times \frac{A_{K}}{a_{s}}, \end{array}$$

where *a*_*k*_ and *a*_*s*_ are the slopes of the analyte and the internal reference standard calibration equations, respectively. *f*_*ks*_ is the average relative conversion factor of each analyte to IRS. *A*_*K*_ is the peak area of the analyte in the pneumonia mixture. *C*_*K*_ is the mass concentration of the analyte in the pneumonia mixture (mg/mL). *n* is the dilution multiple of pneumonia mixture in preparation of the sample solution.

#### 2.6.2. Positioning of the Investigated Components [29, 39]

The position of the selected internal reference standard (IRS) can be accurately and directly identified by using its reference substance. The relative retention time (RRT) has been usually used to locate the chromatographic peaks of the analytes by calculating the ratios of their retention time to that of IRS equation:

(8) $$\begin{array}{c} t_{ks}=\frac{t_{k}}{t_{s}}, \end{array}$$

where *t*_*s*_ is the retention time of internal reference standard and *t*_*k*_ is the retention time of the analyte.

#### 2.6.3. Selection of the Internal Reference Standard

According to the rules of QAMS, it was very important to choose an appropriate internal reference standard, which is active, stable, easily obtainable, cheap, and high-content in sample [39]. The mixed standard solutions were used for the evaluation of the accuracy of QAMS method by standard method difference (SMD) calculated according to the following equation [29]:

(9) $$\begin{array}{c} \text{SMD}=\frac{\left|C_{ES}-C_{\text{QAMS}}\right|}{C_{ES}}\times 100\%, \end{array}$$

where *C*_*ES*_ and *C*_QAMS_ represent the concentrations of an analyte assayed by the external standard method and QAMS method, respectively.

In this paper, all of the eight components could be regarded as IRS to calculate the SMDs, which were also the important basis for the selection of the IRS. And the AVG and LRG method were used to calculate the RCFs of analytes with different IRSs according to (4) and (6), respectively.

### 2.7. Robustness of the QAMS Method

For further investigation on the robustness of QAMS method, the mixed standard solution should be analyzed under different condition. The RCFs and RTT of the other target peaks to IRS were calculated in different flow rates, column temperatures, and columns using the AVG method and assessed by relative standard deviation (RSD, less than 5%) [39]. The flow rate was set at 0.3/0.4/0.5 mL/min with temperature maintained at 30/35/40°C, respectively. Waters ACQUITY UPLC® BEH C18 column (2.1 × 50 mm, 1.7 *μ*m), Waters ACQUITY UPLC® BEH C18 column (2.1 × 100 mm, 1.7 *μ*m), SHIMADZU Shim-pack GIST C18-HP column (50 mm × 2.1 mm, 3 *μ*m), SHIMADZU Shim-pack GIST C18-HP column (100 mm × 2.1 mm, 3 *μ*m), and SHIMADZU Shim-pack GIST C18-HP column (150 mm × 2.1 mm, 3 *μ*m) were used to study the effect of different columns.

### 2.8. Assessment of the QAMS Method and External Standard Method (ESM)

Sample solutions of pneumonia mixture were used for the evaluation of the deviation value of QAMS method. The contents of eight constituents in pneumonia mixture were directly determined by using the validated ESM. With the results of RCFs, the content of eight constituents except IRS in the samples could be indirectly calculated by AVG and LRG method according to (5) and (7), respectively. The concentrations of eight constituents calculated through QAMS method were compared to the calculative concentration of sample solution through the classic ESM to assess the similarity of QAMS method and ESM and to verify the feasibility of QAMS by F-test using SPSS 17.0 and standard method difference (SMD).

## 3. Results and Discussion

### 3.1. Method Validation

#### 3.1.1. Specificity

**Figure 2:**
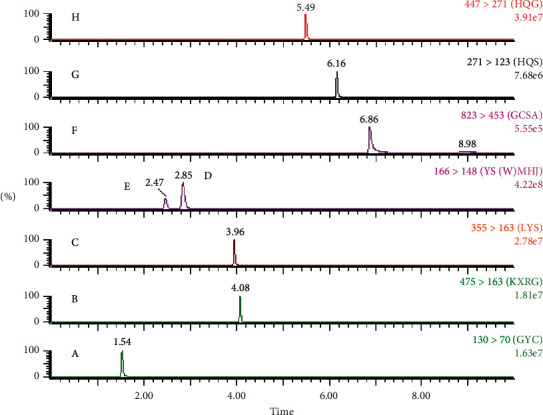
Typical UPLC-MS/MS analysis MRM chromatograms of the mixed standard solution: A (R, S)-goitrin, B amygdalin, C chlorogenic acid, D pseudoephedrine hydrochloride, E ephedrine hydrochloride, F ammonium glycyrrhizinate, G baicalein, and H baicalin.

**Figure 3:**
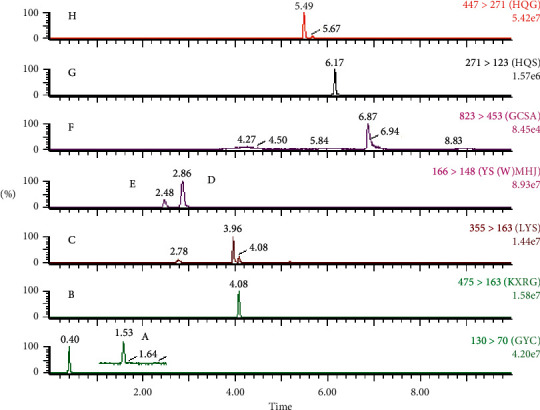
Typical UPLC-MS/MS analysis MRM chromatograms of the sample solution: A (R, S)-goitrin, B amygdalin, C chlorogenic acid, D pseudoephedrine hydrochloride, E ephedrine hydrochloride, F ammonium glycyrrhizinate, G baicalein, and H baicalin.

The typical chromatograms of the mixed standard and sample solutions are shown in Figures 2 and 3. No significant endogenous interference was found in the retention times of pseudoephedrine hydrochloride (2.85 min), ephedrine hydrochloride (2.47 min), amygdalin (4.08 min), baicalein (6.16 min), baicalin (5.49 min), (R, S)-goitrin (1.54 min), chlorogenic acid (3.96 min), and ammonium glycyrrhizinate (6.86 min).

#### 3.1.2. Linearity

The calibration curves and correlation coefficients of the eight analytes are shown in Table 1. The correlation coefficients (*r* > 0.9990) of the calibration curves exhibited good linearity over the selected concentration ranges.

#### 3.1.3. Precision

The relative standard deviations (RSDs) for intra- and interday precisions of all the eight analytes were lower than 4.77% and 4.83%, which were within the acceptable limits. The results of precision are presented in Table S2, suggesting that the method had a good precision for the determination of the eight analytes in pneumonia mixture.

#### 3.1.4. Stability

The RSDs of the peak areas of the eight components at various time were within the range 1.98–4.72%, indicating that the sample solution was stable for 12 h at autosampler (15 °C) and the method established was reliable. The results are summarized in Table S2.

#### 3.1.5. Repeatability

The RSD values of the peak areas of the eight analytes were in the range of 1.37–5.96%, indicating that the method is reproducible. The results of repeatability are shown in Table S2.

#### 3.1.6. Accuracy

As shown in Table S3, the average recoveries of the eight active components in pneumonia mixture at three concentration levels were satisfactory, with values in the range of 85.25% to 113.61% and RSDs less than 8.74%. The results show that the established method is accurate enough to determine the eight active components in the sample.

### 3.2. Calculation of Relative Conversion Factors and Relative Retention Time of the QAMS Method

**Figure 4:**
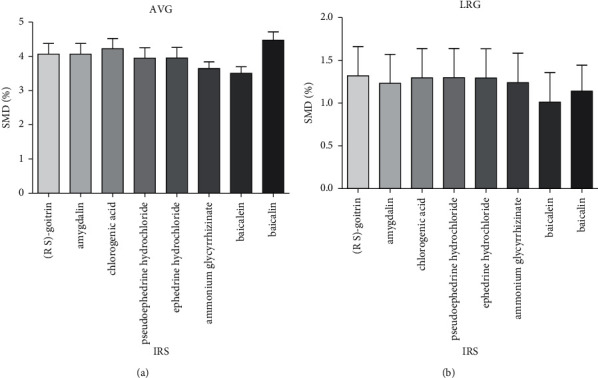
Bar charts of the SMDs. The RCFs were calculated by AVG (a) and LRG (b) methods.

For a more intuitive description of the data of selecting internal reference standard, bar charts of the SMDs were prepared, as shown in Figure 4. In Figure 4(a), the RCFs were calculated by the AVG method, and in Figure 4(b), the RCFs were calculated by LRG method. There were no significant differences (*P* > 0.05) in SMDs among all the eight components. The results of SMDs indicated that all of the components could be regarded as IRSs to calculate the results, of which the SMDs were found to be ＜5%. In this paper, we selected cheap, easily obtainable, high-content, and chemically stable baicalein with the lowest SMDs as an internal reference standard for the quantitative determination of other active components. In addition to that, baicalein is one of the main active ingredients of *Scutellaria baicalensis* Georgi [40] and can be easily separated under the conventional chromatographic conditions, so baicalein was taken as an internal reference standard in this study. The RCFs of 7 components in pneumonia mixture were calculated using the AVG and LRG method, respectively. The values of *t*_*ks*_ were obtained according to (8). The data in Table 2 show the RCFs and RRT of (R, S)-goitrin, amygdalin, chlorogenic acid, pseudoephedrine hydrochloride, ephedrine hydrochloride, ammonium glycyrrhizinate, and baicalin to baicalein. The results of the LRG method were similar to those of the AVG method and were considered to be accurate and stable. It can be seen that the RCFs of 7 components were in the range of 0.07 to 158.22, which clearly indicates the dependence of the signal intensity on the structure. In case of MS detection in MRM mode, the differences among UV absorption features of eight constituents in pneumonia mixture were not considered and the position of the analytes were identified and located by the RRT combined with the exact masses of precursor and fragment ions and their abundance according to the method given in work [32, 41].

### 3.3. Robustness of the QAMS Method

As shown in Tables S4–S6, with the change of chromatographic conditions, the value of RCFs almost stays the same, and the RSDs of RCFs calculated in different flow rates, column temperatures, and columns turned out acceptable, which illustrated the notion that RCFs values had good repeatability and were acceptable for quantitative analysis. The RSDs of the RTT of (R, S)-goitrin, pseudoephedrine hydrochloride, and ephedrine hydrochloride in different flow rates and columns were within the range 13.94–34.86%, indicating that the relative retention time only cannot identify the chromatographic peak position of the three analytes in the circumstances. The position of the three analytes can be identified and located by the relative retention time combined with the exact masses of precursor and fragment ions and their abundance. The RSDs of the RTT of amygdalin, chlorogenic acid, ammonium glycyrrhizinate, and baicalin in different flow rates and columns were lower than 4.09% and those of all the seven components in different column temperatures were lower than 4.93%, indicating that the relative retention time was stable and could identify the chromatographic peak position of the four analytes.

### 3.4. Assessment of the QAMS Method and External Standard Method (ESM)

The concentrations of eight constituents in pneumonia mixture were obtained by ESM, AVG, and LRG method, separately, seen in Table 3. All SMDs were found to be ＜ 9.02%, which showed that the content of (R, S)-goitrin, amygdalin, chlorogenic acid, pseudoephedrine hydrochloride, ephedrine hydrochloride, ammonium glycyrrhizinate, and baicalin obtained by LRG and AVG method had good similarity to that calculated by ESM. And SMDs of LRG method were mostly lower than those of AVG method. There were no significant differences (*P* > 0.05) in the content results of all the seven components between ESM and LRG methods. There were significant differences in the content results of amygdalin (*P* < 0.01), pseudoephedrine hydrochloride (P < 0.01), ephedrine hydrochloride (*P* < 0.05), and baicalin (*P* < 0.01) between ESM and AVG methods. All these results demonstrated that both LRG and AVG method had good accuracy and were stable, feasible, and credible to quantitative assay of eight constituents in pneumonia mixture. But LRG method had higher accuracy in comparison with AVG method. What is the reason for this difference? The data with relatively large deviation might cause larger effect on RCFs in AVG method than that in LRG method. So, LRG method was much more accurate and stable than AVG method for the calculation of RCFs in QAMS method.

## 4. Conclusion

Only a few ingredients of the standard determination of content could not control the quality of pneumonia mixture effectively. It is necessary to use multiple active ingredients as index components to control the quality of pneumonia mixture preparations more effectively and comprehensively. In the present study, a quantitative analysis of multicomponent via a single marker (QAMS) method was developed for the simultaneous quantitative analysis of eight active components of pneumonia mixture by UPLC-MS/MS. After calculating the RCFs of other seven components by AVG and LRG method, of which baicalein was taken as the IRS, and validation of QAMS method, the contents of eight active components were obtained. For comparison, the content of eight active components was also determined by a classic external standard method. The contents of each target compounds were stable and similar in ESM, AVG, and LRG method, indicating that QAMS method possessed high accuracy and feasibility. The QAMS method did not require preparation for all the eight reference standards solutions; instead, only baicalein was used in further measurements, which reduces analytical cost and time of detection and avoids the problem of the diversity and large quantity of reference standards, some of which are expensive or unstable. Our findings were conclusive proof to the practicability of the application of QAMS method for the quantitative analysis of multicomponents in pneumonia mixture or even a group of similar medicines.

## Figures and Tables

**Table 1:** Linear regression data of the eight analytes.

Analytes	Calibration curves	Correlation coefficients (r)
(R, S)-goitrin	*y* = 7449.48*x* + 168.691	0.9997
Amygdalin	*y* = 380.53*x* + 6102.67	0.9992
Chlorogenic acid	*y* = 1152.30*x* + 239.22	0.9995
Pseudoephedrine hydrochloride	*y* = 84605.00*x* + 7019.50	0.9990
Ephedrine hydrochloride	*y* = 41945.40*x* + 3473.01	0.9990
Ammonium glycyrrhizinate	*y* = 74.41*x* + 73.66	0.9993
Baicalein	*y* = 616.17*x* − 146.83	0.9996
Baicalin	*y* = 888.61*x* + 23186.00	0.9995

**Table 2:** RCFs and RRT of all the seven components to baicalein.

Analytes	*f*_*ks*_		*t*_*ks*_
	AVG method	LRG method	
(R, S)-goitrin	12.98 ± 0.86	12.09	0.25
Amygdalin	0.68 ± 0.05	0.62	0.66
Chlorogenic acid	1.98 ± 0.10	1.87	0.64
Pseudoephedrine hydrochloride	148.62 ± 7.51	137.31	0.46
Ephedrine hydrochloride	73.69 ± 1.82	68.07	0.40
Ammonium glycyrrhizinate	0.12 ± 0.01	0.12	1.12
Baicalin	1.60 ± 0.10	1.44	0.89

**Table 3:** Contents of the eight components in the pneumonia mixture determined by ESM, AVG, and LRG methods.

Sample	Baicalein	(R, S)-goitrin					Amygdalin					Chlorogenic acid				
	ESM (mg/mL)	ESM (mg/mL)	AVG (mg/mL)	SMD (%)	LRG (mg/mL)	SMD (%)	ESM (mg/mL)	AVG## (mg/mL)	SMD (%)	LRG (mg/mL)	SMD (%)	ESM (mg/mL)	AVG (mg/mL)	SMD (%)	LRG (mg/mL)	SMD (%)

1	0.47	0.0045	0.0043	3.81	0.0045	0.27	7.41	7.05	4.89	7.46	0.69	2.02	1.97	2.68	2.02	0.04
2	0.44	0.0040	0.0042	4.59	0.0044	9.02	7.46	7.10	4.90	7.51	0.68	2.07	2.02	2.67	2.08	0.06
3	0.45	0.0040	0.0041	3.41	0.0043	7.79	7.45	7.09	4.90	7.50	0.68	2.10	2.04	2.67	2.10	0.06
4	0.42	0.0040	0.0042	4.37	0.0044	8.80	7.20	6.85	4.86	7.25	0.72	1.99	1.94	2.66	2.00	0.06
5	0.45	0.0045	0.0044	1.65	0.0046	2.52	7.31	6.96	4.89	7.37	0.69	2.09	2.03	2.67	2.09	0.05
6	0.43	0.0040	0.0040	0.36	0.0042	4.61	7.17	6.82	4.86	7.22	0.72	1.98	1.93	2.66	1.99	0.06

Sample	Pseudoephedrine hydrochloride					Ephedrine hydrochloride					Ammonium glycyrrhizinate					Baicalin				
	ESM (mg/mL)	AVG## (mg/mL)	SMD (%)	LRG (mg/mL)	SMD (%)	ESM (mg/mL)	AVG# (mg/mL)	SMD (%)	LRG (mg/mL)	SMD (%)	ESM (mg/mL)	AVG (mg/mL)	SMD (%)	LRG (mg/mL)	SMD (%)	ESM (mg/mL)	AVG## (mg/mL)	SMD (%)	LRG (mg/mL)	SMD (%)

1	0.39	0.37	4.68	0.39	0.17	0.18	0.17	4.66	0.18	0.20	0.49	0.52	4.53	0.50	0.85	11.55	10.85	6.02	11.68	1.13
2	0.39	0.37	4.78	0.39	0.06	0.17	0.17	4.53	0.18	0.33	0.42	0.44	4.69	0.42	1.00	11.59	10.89	6.02	11.72	1.13
3	0.40	0.38	4.80	0.40	0.04	0.18	0.17	4.65	0.18	0.21	0.42	0.44	4.72	0.42	1.03	11.64	10.94	6.03	11.77	1.12
4	0.38	0.36	4.75	0.38	0.10	0.17	0.16	4.68	0.17	0.18	0.42	0.44	4.76	0.43	1.07	11.23	10.56	5.99	11.36	1.16
5	0.39	0.37	4.76	0.39	0.08	0.18	0.17	4.61	0.18	0.25	0.39	0.41	4.79	0.40	1.10	11.59	10.89	6.02	11.72	1.13
6	0.38	0.36	4.73	0.38	0.12	0.17	0.16	4.50	0.17	0.37	0.40	0.42	4.78	0.41	1.09	11.40	10.71	6.01	11.53	1.14

^#^ *P* < 0.05 and ^##^*P* < 0.01, AVG group vs. ESM group.

## Data Availability

The data used to support the findings of this study are included within the article.

## Supplementary Materials

Supplementary Materials Figure S1: MS/MS scan spectra of (R, S)-goitrin, amygdalin, chlorogenic acid, pseudoephedrine hydrochloride, ephedrine hydrochloride, ammonium glycyrrhizinate, baicalein, and baicalin. Table S1: optimized multiple reaction monitoring (MRM) parameters for eight analytes. Table S2: intra- and interday precision, stability, and reproducibility of the developed method. Table S3: recoveries of the eight analytes in the pneumonia mixture. Table S4: the values of *f*_*ks*_ and *t*_*ks*_ calculated with different flow rates. Table S5: the values of *f*_*ks*_ and *t*_*ks*_ calculated with different column temperatures. Table S6: the values of *f*_*ks*_ and *t*_*ks*_ calculated with different columns.
