# Supplementary material for: Systematic Study on a Quantitative Analysis of Multicomponents by Single Marker (QAMS) Method for Simultaneous Determination of Eight Constituents in Pneumonia Mixture by UPLC-MS/MS
Source: J Anal Methods Chem. 2021 Nov 3;2021:8311588. doi: 10.1155/2021/8311588 (PMC8580625; doi:10.1155/2021/8311588)
Supplement: Supplementary Materials — Figure S1: MS/MS scan spectra of (R, S)-goitrin, amygdalin, chlorogenic acid, pseudoephedrine hydrochloride, ephedrine hydrochloride, ammonium glycyrrhizinate, baicalein, and baicalin. Table S1: optimized multiple reaction monitoring (MRM) parameters for eight analytes. Table S2: intra- and interday precision, stability, and reproducibility of the developed method. Table S3: recoveries of the eight analytes in the pneumonia mixture. Table S4: the values of fks and tks calculated with different flow rates. Table S5: the values of fks and tks calculated with different column temperatures. Table S6: the values of fks and tks calculated with different columns. [file 8311588.f1.doc]

**Supporting Information**

**Systematic study on a quantitative analysis of multi-components by single marker (QAMS) method for simultaneously determination of eight constituents in pneumonia mixture by UPLC-MS/MS**

Haibo Zhang,^1,2^ Weina Xie,^1,2^ Jiangyun Liu,^3^ Xiaoqiang Xiang,^4^ Shilei Zhang,^1^ Junping Hu,^1^ Jianhua Yang^1,2^

^1^ College of Pharmacy, Xinjiang Medical University, Urumqi 830011, PR China

^2^ Department of Pharmacy, The First Affiliated Hospital of Xinjiang Medical University, Urumqi 830011, PR China

^3^ College of Pharmaceutical Sciences, Soochow University, Suzhou 215123, PR China

^4^ Department of Clinical Pharmacy, School of Pharmacy, Fudan University, Shanghai 201203, PR China

* Corresponding author.

mail addresses:89229613@qq.com (Junping Hu), 609571241@qq.com (Jianhua Yang)

Weina Xie is the co-first author.

**Table S1 Optimized multiple reaction monitoring (MRM) parameters for eight analytes.**

| Analyte | Parent（m/z） | Daughter（m/z） | Cone（V） | Collision（V） |
| --- | --- | --- | --- | --- |
| (R, S)-goitrin | 129.9 | 70.2 | 15 | 10 |
| amygdalin | 475.1 | 162.8 | 15 | 15 |
| chlorogenic acid | 354.9 | 163.1 | 25 | 10 |
| pseudoephedrine hydrochloride | 165.9 | 148.0 | 15 | 10 |
| ephedrine hydrochloride | 165.9 | 148.0 | 15 | 10 |
| ammonium glycyrrhizinate | 822.9 | 453.1 | 10 | 35 |
| baicalein | 270.9 | 122.9 | 15 | 35 |
| baicalin | 447.1 | 270.9 | 25 | 15 |

**Table S2 Intra- and inter-day precision, stability and reproducibility of the developed method.**

| Analytes | Precision (RSD, %) | | Stability  (RSD, %) (n=6) | Reproducibility  (RSD, %)  (n=6) |
| --- | --- | --- | --- | --- |
|  | Intra-day  (n=6) | Inter-day  (n=3) |  |  |
| (R, S)-goitrin | 0.59 | 1.29 | 4.07 | 5.96 |
| amygdalin | 0.82 | 0.48 | 2.24 | 2.11 |
| chlorogenic acid | 2.01 | 4.83 | 3.79 | 2.37 |
| pseudoephedrine hydrochloride | 0.42 | 1.53 | 1.98 | 2.22 |
| ephedrine hydrochloride | 0.70 | 1.57 | 2.26 | 1.57 |
| ammonium glycyrrhizinate | 4.77 | 4.10 | 3.94 | 3.18 |
| baicalein | 0.84 | 2.69 | 2.14 | 3.26 |
| baicalin | 1.58 | 4.52 | 4.72 | 1.37 |

**Table S3 Recoveries of the eight analytes in pneumonia mixture (n=5).**

| Analytes | concentration level | Average recovery (%) | RSD (%) |
| --- | --- | --- | --- |
| (R, S)-goitrin | low | 95.56 | 5.60 |
|  | medium | 104.44 | 8.74 |
|  | high | 107.41 | 0.00 |
| amygdalin | low | 106.73 | 0.96 |
|  | medium | 91.66 | 2.78 |
|  | high | 88.46 | 1.90 |
| chlorogenic acid | low | 97.76 | 2.06 |
|  | medium | 85.25 | 4.29 |
|  | high | 85.47 | 1.29 |
| pseudoephedrine hydrochloride | low | 113.61 | 2.22 |
|  | medium | 106.81 | 3.51 |
|  | high | 108.36 | 1.16 |
| ephedrine hydrochloride | low | 105.38 | 2.96 |
|  | medium | 98.35 | 2.87 |
|  | high | 101.96 | 1.05 |
| ammonium glycyrrhizinate | low | 98.04 | 2.76 |
|  | medium | 98.06 | 5.11 |
|  | high | 102.61 | 2.89 |
| baicalein | low | 106.89 | 8.58 |
|  | medium | 100.22 | 3.42 |
|  | high | 107.57 | 3.36 |
| baicalin | low | 103.05 | 2.28 |
|  | medium | 97.35 | 2.64 |
|  | high | 96.97 | 1.17 |

**Table S4 The values of $f_{ks}$ and $t_{ks}$ calculated with different flow rate（n=3）**

| flow rate（mL/min） | (R, S)-goitrin | | amygdalin | | chlorogenic acid | | pseudoephedrine hydrochloride | | ephedrine hydrochloride | | ammonium glycyrrhizinate | | baicalin | |
| --- | --- | --- | --- | --- | --- | --- | --- | --- | --- | --- | --- | --- | --- | --- |
|  | $f_{ks}$ | $t_{ks}$ | $f_{ks}$ | $t_{ks}$ | $f_{ks}$ | $t_{ks}$ | $f_{ks}$ | $t_{ks}$ | $f_{ks}$ | $t_{ks}$ | $f_{ks}$ | $t_{ks}$ | $f_{ks}$ | $t_{ks}$ |
| 0.3 | 12.39 | 0.32 | 0.69 | 0.68 | 2.04 | 0.66 | 144.90 | 0.56 | 75.60 | 0.51 | 0.11 | 1.10 | 1.56 | 0.89 |
| 0.4 | 12.98 | 0.25 | 0.68 | 0.66 | 1.98 | 0.64 | 148.62 | 0.46 | 73.69 | 0.40 | 0.12 | 1.12 | 1.60 | 0.89 |
| 0.5 | 13.30 | 0.21 | 0.66 | 0.65 | 1.85 | 0.63 | 149.59 | 0.39 | 78.84 | 0.33 | 0.12 | 1.12 | 1.51 | 0.89 |
| Mean | 12.89 | 0.26 | 0.68 | 0.66 | 1.96 | 0.64 | 147.70 | 0.47 | 76.04 | 0.42 | 0.12 | 1.11 | 1.56 | 0.89 |
| RSD（%） | 3.59 | 21.00 | 2.35 | 2.24 | 4.96 | 2.68 | 1.68 | 17.86 | 3.43 | 21.38 | 4.95 | 0.86 | 2.90 | 0.15 |

**Table S5 The values of $f_{ks}$ and $t_{ks}$ calculated with different column temperature（n=3）**

| column temperature（℃） | (R, S)-goitrin | | amygdalin | | chlorogenic acid | | pseudoephedrine hydrochloride | | ephedrine hydrochloride | | ammonium glycyrrhizinate | | baicalin | |
| --- | --- | --- | --- | --- | --- | --- | --- | --- | --- | --- | --- | --- | --- | --- |
|  | $f_{ks}$ | $t_{ks}$ | $f_{ks}$ | $t_{ks}$ | $f_{ks}$ | $t_{ks}$ | $f_{ks}$ | $t_{ks}$ | $f_{ks}$ | $t_{ks}$ | $f_{ks}$ | $t_{ks}$ | $f_{ks}$ | $t_{ks}$ |
| 30 | 12.98 | 0.25 | 0.68 | 0.66 | 1.98 | 0.64 | 148.62 | 0.46 | 73.69 | 0.40 | 0.12 | 1.12 | 1.60 | 0.89 |
| 35 | 12.87 | 0.25 | 0.63 | 0.66 | 1.92 | 0.64 | 140.23 | 0.45 | 74.94 | 0.39 | 0.11 | 1.12 | 1.56 | 0.89 |
| 40 | 13.48 | 0.23 | 0.66 | 0.66 | 1.97 | 0.63 | 151.75 | 0.42 | 79.46 | 0.37 | 0.11 | 1.12 | 1.52 | 0.89 |
| Mean | 13.11 | 0.24 | 0.66 | 0.66 | 1.96 | 0.64 | 146.87 | 0.45 | 76.03 | 0.39 | 0.11 | 1.12 | 1.56 | 0.89 |
| RSD（%） | 2.50 | 3.82 | 3.48 | 0.40 | 1.64 | 1.37 | 4.06 | 4.93 | 3.99 | 4.44 | 5.09 | 0.31 | 2.56 | 0.20 |

**Table S6 The values of $f_{ks}$ and $t_{ks}$ calculated with different column（n=3）**

| column | (R, S)-goitrin | | amygdalin | | chlorogenic acid | | pseudoephedrine hydrochloride | | ephedrine hydrochloride | | ammonium glycyrrhizinate | | baicalin | |
| --- | --- | --- | --- | --- | --- | --- | --- | --- | --- | --- | --- | --- | --- | --- |
|  | $f_{ks}$ | $t_{ks}$ | $f_{ks}$ | $t_{ks}$ | $f_{ks}$ | $t_{ks}$ | $f_{ks}$ | $t_{ks}$ | $f_{ks}$ | $t_{ks}$ | $f_{ks}$ | $t_{ks}$ | $f_{ks}$ | $t_{ks}$ |
| Waters 50 | 12.98 | 0.25 | 0.68 | 0.66 | 1.98 | 0.64 | 148.62 | 0.46 | 73.69 | 0.40 | 0.12 | 1.12 | 1.60 | 0.89 |
| Waters 100 | 13.24 | 0.44 | 0.63 | 0.69 | 1.80 | 0.68 | 144.69 | 0.61 | 71.91 | 0.60 | 0.13 | 1.08 | 1.63 | 0.89 |
| SHIMADZU 50 | 12.06 | 0.31 | 0.67 | 0.66 | 1.94 | 0.66 | 149.73 | 0.49 | 75.11 | 0.43 | 0.12 | 1.08 | 1.73 | 0.89 |
| SHIMADZU 100 | 13.60 | 0.54 | 0.65 | 0.69 | 1.85 | 0.69 | 141.96 | 0.61 | 75.25 | 0.59 | 0.11 | 1.05 | 1.71 | 0.89 |
| SHIMADZU 150 | 13.14 | 0.61 | 0.69 | 0.71 | 1.83 | 0.72 | 148.90 | 0.63 | 73.56 | 0.63 | 0.11 | 1.04 | 1.64 | 0.90 |
| Mean | 13.00 | 0.43 | 0.66 | 0.68 | 1.88 | 0.68 | 146.78 | 0.56 | 73.90 | 0.53 | 0.12 | 1.07 | 1.66 | 0.89 |
| RSD（%） | 4.42 | 34.86 | 3.61 | 3.09 | 4.07 | 4.09 | 2.26 | 13.94 | 1.84 | 19.88 | 7.09 | 2.73 | 3.33 | 0.52 |

**
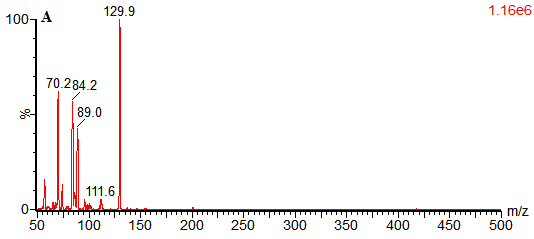
**

**
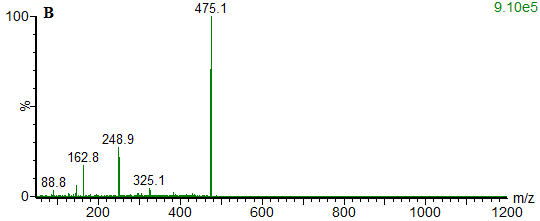

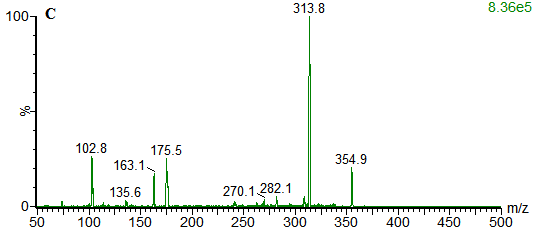

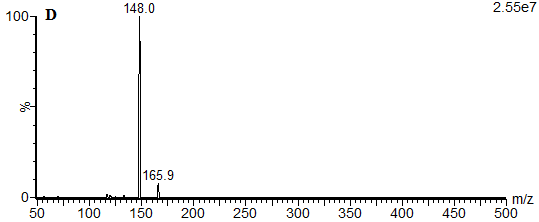

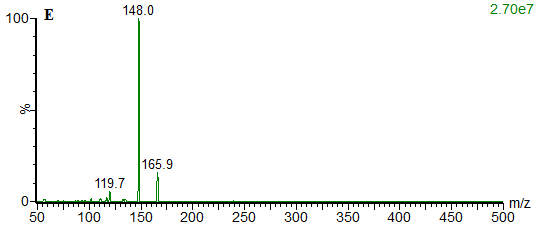

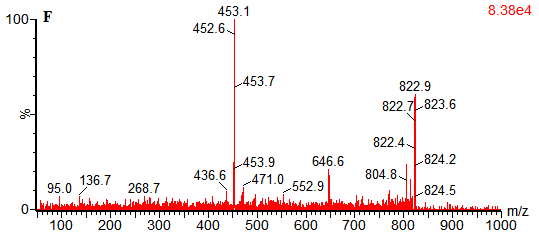

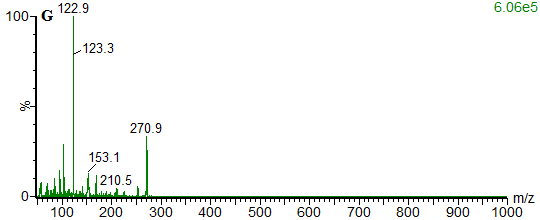

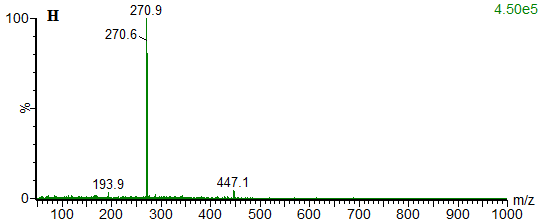
**

**Fig. S1 MS/MS scan spectrums of (A) (R, S)-goitrin, (B) amygdalin, (C) chlorogenic acid, (D) pseudoephedrine hydrochloride, (E) ephedrine hydrochloride, (F) ammonium glycyrrhizinate, (G) baicalein, (H) baicalin**
